# Acute effect of propranolol on resting energy expenditure in hyperthyroid patients

**DOI:** 10.3389/fendo.2022.1026998

**Published:** 2023-01-19

**Authors:** Jaël Rut Senn, Rahel Catherina Löliger, Jonas Gabriel William Fischer, Fabienne Bur, Claudia Irene Maushart, Matthias Johannes Betz

**Affiliations:** ^1^ Department of Endocrinology, Diabetes and Metabolism, University Hospital Basel, Basel, Switzerland; ^2^ Faculty of Medicine, University of Basel, Basel, Switzerland

**Keywords:** hyperthyroidism, thyroid hormone, energy expenditure, propranolol, brown adipose tissue (BAT)

## Abstract

**Objective:**

Hyperthyroidism is a common endocrine disorder which leads to higher resting energy expenditure (REE). Increased activity of brown adipose tissue (BAT) contributes to elevated REE in hyperthyroid patients. For rapid control of hyperthyroid symptoms, the non-selective β-blocker propranolol is widely used. While, long-term treatment with propranolol reduces REE it is currently unclear whether it can also acutely diminish REE.

**Design:**

In the present prospective interventional trial we investigated the effect of propranolol on REE in hyperthyroid patients.

**Methods:**

Nineteen patients with overt primary hyperthyroidism were recruited from the endocrine outpatient clinic. REE was measured by indirect calorimetry before and after an acute dose of 80mg propranolol and during a control period, respectively. Additionally, skin temperature was recorded at eleven predefined locations during each study visit, vital signes and heart rate (HR) were measured before and after administration of propranolol.

**Results:**

Mean REE decreased slightly after acute administration of 80mg propranolol (p= 0.03) from 1639 ± 307 kcal/24h to 1594 ± 283 kcal/24h. During the control visit REE did not change significantly. HR correlated significantly with the level of free T3 (R^2^ = 0.38, p=0.029) free T4 (R^2^ = 0.39, p=0.026). HR decreased 81 ± 12 bpm to 67 ± 7.6 bpm 90 minutes after oral administration of propranolol (p<0.0001). Skin temperature did not change after propranolol intake.

**Conclusions:**

In hyperthyroid patients a single dose of propranolol reduced heart rate substantially but REE diminished only marginally probably due to reduced myocardial energy consumption. Our data speak against a relevant contribution of BAT to the higher REE in hyperthyroidism.

**Clinical trial registration:**

ClinicalTrials.gov, identifier (NCT03379181).

## 1 Introduction

Hyperthyroidism is a common endocrine disorder, which is characterized by an excessive synthesis and release of thyroid hormones (TH). Hyperthyroid patients often suffer from weight loss and heat intolerance due to higher resting energy expenditure (REE) (1–3). Excess levels of TH increase REE *via* peripheral and central mechanisms. In skeletal muscle TH increase the transcription of sarcoplasmic reticulum Ca^2+^-ATPase which increases skeletal muscle respiration and energy expenditure (4). Centrally, TH activates the sympathetic nervous system (SNS) *via* the ventromedial nucleus of the hypothalamus (5).

The SNS and its main transmitter norepinephrine activate brown adipose tissue (BAT). BAT is a thermogenic tissue which is usually activated in response to cold exposure in order to maintain the normal body core temperature. It can convert chemical energy from triglycerides or glucose directly into heat. Brown adipocytes contain a large number of mitochondria and store lipids in small intracellular droplets as opposed to the large single lipid drop found in white adipocytes.

The importance of TH for the function and differentiation of brown adipocytes is underscored by the fact that BAT expresses high levels of deiodinase 2 (DIO2) which converts thyroxin (T4) to triiodothyronine (T3) (6). Research in rodents (7) and humans (8) revealed that hyperthyroidism activates BAT in the absence of cold. Thus, higher BAT activity could contribute partially to the higher REE in humans suffering from hyperthyroidism.

Medical treatment of hyperthyroidism aims to reduce TH synthesis and to lower plasma TH levels using antithyroid drugs such as methimazole. However, β-adrenoreceptor (β-AR) antagonists (β-blockers) are often administered to rapidly control hyperthyroid symptoms. Especially the non-selective β-blocker propranolol is widely used and is considered the drug of choice (9–11). In humans, treatment with propranolol over the course of four weeks reduced REE while treatment with the β_1_-selective antagonist metoprolol did not (12–14). However, the acute effects of β-blockers on REE in patients with hyperthyroidism are currently unclear. Several interventional studies demonstrated a reduction of cold-induced BAT activity in ^18^F-FDG-PET/CT scans after blocking the β-ARs with propranolol in euthyroid patients (15–17).

We hypothesized that propranolol might immediately decrease REE in patients suffering from primary hyperthyroidism by reducing BAT activity. We investigated the effect of a single dose of 80 mg of propranolol on REE in hyperthyroid patients.

## 2 Subjects and methods

### 2.1 Subjects

From January 2018 to February 2020 we enrolled 19 patients between 20 to 70 years of age presenting to the outpatient endocrine clinic at the University Hospital Basel, Switzerland. Patients were eligible if the TSH value was below 0.2 mlU/l and the level of free T4 at or above 25 pmol/l or free T3 at or above 8 pmol/l. Patients with a BMI above 30 kg/m^2^, pregnant or breastfeeding women, uncontrolled diabetes (HbA1c >7.5%), asthma, chronic obstructive pulmonary disease or any other significant chronic or acute disease such as heart or kidney failure, liver cirrhosis or metastasized cancer, abuse of alcohol or illicit drugs, prolonged electrographic PR interval or pre-existing therapy with beta-blockers or antithyroid medication were excluded.

The study protocol was reviewed and approved on December 11, 2017, by the medical ethics committee of the University of Basel (ID EKNZ 2017-02044) and registered on ClinicalTrials.gov (NCT03379181). All participants provided written informed consent.

### 2.2 Study design

This was a prospective interventional trial to assess the effect of a single dose propranolol on REE in hyperthyroid patients. In all participants REE was measured before and 90 minutes after a sigle dose of 80mg propranolol (Treatment). To exclude a significant influence of resting period between the two measurements *per se*, a control visit without a study drug administration was introduced after the first eleven patients had been recruited (Control). This control visit was performed in a randomized order to the intervention visit in the last eight patients.

The study visits and examinations took place in the morning, after a fasting period of at least 6 hours. The study comprised also an observational part in which the effect of hyperthyroidism on cold-induced thermogenesis (CIT) and body composition were evaluated and which has been published previously (18).

### 2.3 Clinical parameters

In all participants, weight and height were measured and body mass index (BMI) was calculated [kg/m^2^]. An ECG was performed at the start of the study visit, to exclude prolonged PR intervals. Additionally, they completed a hyperthyroid symptom scale (**Supplementary Table 1**). Blood pressure [mmHg] and heart rate (HR) [bpm] were measured at the beginning of the study visit, and heart rate was monitored every 30 minutes after administration of propranolol.

### 2.4 Laboratory parameters

We measured serum thyroid stimulating hormone (TSH), free trijodothyronine (fT3), free thyroxine (fT4) and glycated hemoglobin (HbA1c) in all trial participants. Laboratory analyses were conducted at the central lab of the University Hospital Basel. TSH and fT3/fT4 were measured with electro-chemiluminescence immunoassays (Elecsys, all assays from Roche Diagnostics GmbH, Mannheim, Germany). The reference ranges were as follows: TSH, 0.332–4.490 mIU/l, free T3: 2.6-5.6 pmol/l and free T4 11.6-22.0 pmol/l. HbA1c was measured at the point of care (DCA Vantage, Siemens Healthineers, Erlangen, Germany).

### 2.5 Measurements of energy expenditure and respiratory quotient

REE and respiratory quotient (RQ) and were measured before and 90 minutes after a single oral dose of 80mg propranolol (Treatment) or after an equivalently long resting period without intervention (Control), respectively. We performed indirect calorimetry for 30 min using a ventilated hood calorimeter (Cosmed Quark RMR, Cosmed, Rome, Italy). The patients lay on a hospital bed in the supine position and were covered with a fleece blanket, in order to prevent cold-induced thermogenesis. Measurements were performed in an air-conditioned study room at a controlled ambient temperature of 24°C year round. Patients were asked to fast overnight (at least 6 hours) and to refrain from intensive physical exercise 24h prior to the study visit.

### 2.6 Measurements of body core temperature and skin temperature

The core body temperature was measured by infrared tympanometry (Braun, ThermoScan PRO 6000, Marlborough, MA) after each calorimetry. The skin surface temperature was monitored continuously every minute during the study visits by wireless iButtons (Maxim Integrated, San Jose, CA, United States) placed at 11 defined body locations. Supraclavicular region (right and left), parasternal at the level of the second intercostal space (right and left), umbilicus, mid-thigh (right and left), middle of the lower arm palmar side, finger tip of the 3^rd^ finger of the non-dominant hand, middle of the lower left leg and back of the left foot. An additional sensor logged the ambient temperature at the study location. The temperature data of every location during the last 10 min were averaged and analyzed.

### 2.7 Statistical analysis

Data were analyzed using GraphPad Prism Version 9.3.1 (GraphPad, La Jolla, CA). Continuous data are given as mean ± SD unless stated otherwise. Pairwise comparisons were performed with paired t-tests. A p-value below 0.05 was considered significant. The relation between TH values and HR was modelled using a logarithmic growth equation with a plateau: HR = Y_m_ – (Y_m_ – Y_0_)*e^-k^*^x^, were e *x* represents the TH level. Conditions were constrained for Y_0_ and Y_m_, with Y_0_ > 30 and Y_m_ < 100 in order to reflect physiology of HR.

## 3 Results

### 3.1 Baseline characteristics

We screened all patients attending our outpatient thyroid clinic for eligibility and recruited 19 patients for the study between January 2018 and July 2019. At screening all patients were hyperthyroid with a TSH level below 0.2 mlU/l and free T4 at or above 25 pmol/L or free T3 at or above 8 pmol/L. However, at the timepoint of the first study visit one female patient with Graves’ disease was already in a slightly hypothyroid state. Therefore we excluded this patient from the analysis.

**Figure 1:**
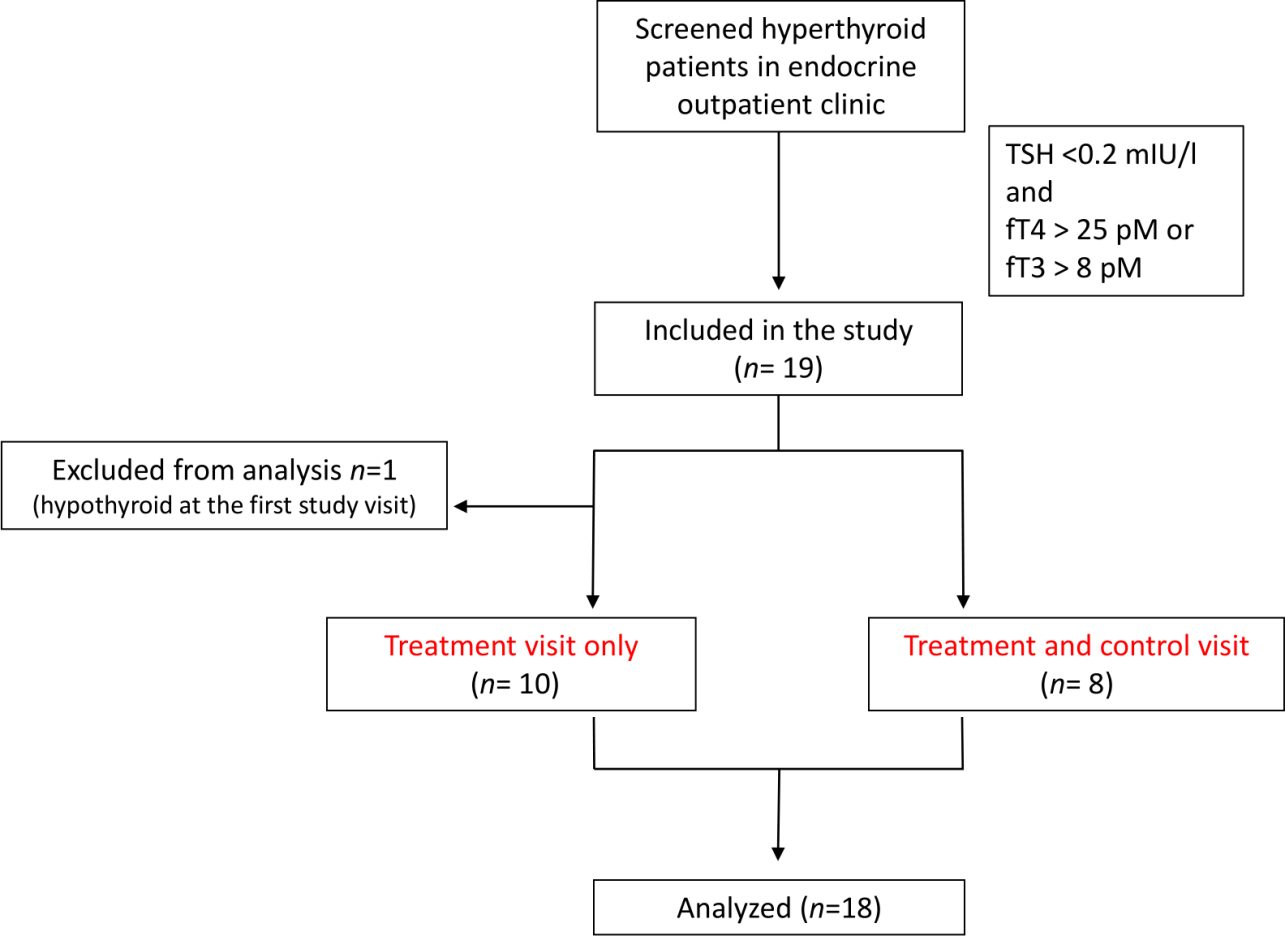
Flow chart of the study population showing included and excluded patients.

Thus, 18 patients (14 female/4 male) were included in the analysis. In the first 10 patients we performed only the interventional treatment visit with propranolol. In the last 8 patients, we conducted the treatment visit and also an additional control visit and the sequence of the visits was randomized (see Study flowchart, **Figure 1**). The two subgroups were comparable in BMI, age and score points on the hyperthyroid symptom scale. Fifteen patients suffered from Graves’ disease, two had thyreoiditis and one had iatrogenic hyperthyroidism. Of these, 15 patients were on thyrostatic therapy at the time of study inclusion. The median of treatment duration was 8 days (see **Supplementary Table 2**).

They were on average 45 ± 17 years old and had a BMI 24 ± 4.5 kg/m². An average score of 14 points was achieved on the hyperthyroidism symptom scale (see **Table 1**).

**Table 1:** Baseline characteristics.

	Group 1 (treatment only) (n=10)	Group 2 (treatment and control) (n=8)	All patients (n=18)	p-value Group 1 vs Group 2
**Age (in years)**	46 ± 17	44 ± 17	45 ± 17	0.73
**Sex**	7 female/3 male	7 female/1 male	14 female/4 male	
**Cause of hyperthyroidism**	- 9 Graves’ disease - 1 overtreatment	- 6 Graves’ disease - 2 subacute thyreoiditis	- 15 Graves’ disease - 2 subacute thyreoiditis - 1 overtreatment	
**Hyperthyroidism symptom scale** **(total points)**	14 ± 8	14 ± 6.0	14 ± 7	0.97
**Weight (kg)**	69 ± 21	66 ± 15	67 ± 18	0.71
**Height (cm)**	170 ± 10	165 ± 7.4	168 ± 9.4	0.26
**BMI (kg/m^2^)**	24 ± 5	24 ± 4	24 ± 4.5	0.80

Pulse rate 0 min Pulse rate 90 min after Propranolol	81 ± 9.2 67 ± 9.5*** p= 0.0007	81 ± 16 67 ± 5.9* p= 0.0137	81 ± 12 67 ± 7.7**** p <0.0001	0.99 0.93
**Systolic blood pressure (mmHg)**	127 ± 18	129 ± 16	128 ± 16	0.74
**Diastolic blood pressure (mmHg)**	74 ± 13	69 ± 7.6	72 ± 11	0.43
**TSH (mlU/L, Ref. 0.332-4.490mIU/l)**	0.0079 ± 0.0078	0.007 ± 0.0042	0.0075 ± 0.0063	0.78
**free T4 (pmol/l, Ref. 11.9-21.6pmol/l)**	38 ± 24	33 ± 9.7	36 ± 18	0.60
**free T3 (pmol/l, Ref. 2.6-5.6pmol/l)**	11 ± 5.6	11 ± 5.3	11 ± 5.3	0.94

REE Before Propranolol REE Before Control	1717 ± 320 -	1541 ± 280 1540 ± 272	1639 ± 307 -	0.24 N/A

*p < 0.05, ***p < 0.001, ****p < 0.0001. N/A= not applicable.

### 3.2 Propranolol and resting energy expenditure

**Figure 2:**
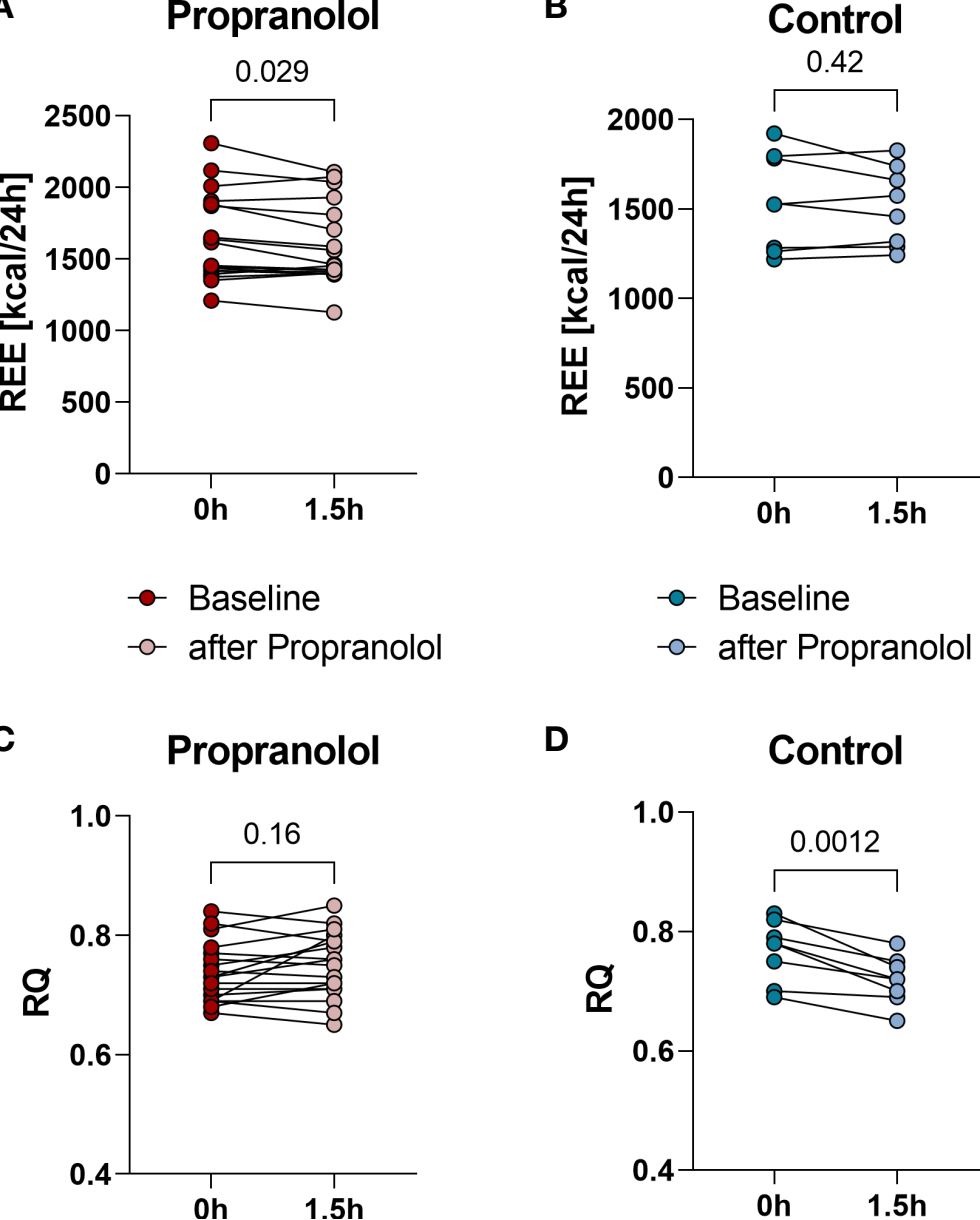
Resting energy expenditure (REE) measured at baseline and 90 minutes after a single dose of 80 mg propranolol or after a 90-minute resting period, respectively. REE decreased significantly (p= 0.029) from baselinie 1639 ± 307 kcal/24h to 1594 ± 283 kcal/24h 90 minutes after administration of propranolol **(A)**. REE at baseline (1540 ± 272 kcal/24h) and after 90- minutes resting period (1513 ± 220 kcal/24h) showed no significant change (p=0.42) in control intervention **(B)**. Respiratory quotient (RQ) at baseline (0.74 ± 0.05) and 90 minutes after Propranolol (0.75 ± 0.055) (p= 0.16) **(C)**. RQ significantly decreased (p=0.0012) from 0.77 ± 0.051 (baseline) to 0.72 ± 0.04 (90 minutes after resting period) during the control intervention **(D)**.

Ninety minutes after a single dose of propranolol mean REE decreased by 45 kcal/24h from 1639 ± 307 kcal/24h to 1594 ± 283 kcal/24h (p=0.029, **Figure 2A**). During the control session, mean REE did not decrease significantly (baseline: 1540 ± 272 kcal/24h, after 90 minutes: 1513 ± 220 kcal/24h, p=0.42, **Figure 2B**). The RQ, an indicator of the amount of carbohydrate or lipids metabolized, remained stable after propranolol (baseline: 0.74 ± 0.05, after 90 minutes: 0.75 ± 0.055, p= 0.16, **Figure 2C**), but decreased significantly after the 90-minutes in the control setting from 0.77 ± 0.051 to 0.72 ± 0.04 (p=0.0012, **Figure 2D**). Additionally we stratified the analysis into the ten patients without a control session (group 1) and the eight patients with a control session (group 2). In group 1 REE decreased from 1717 kcal/24h to 1652 kcal/24h (p=0.01) and in group 2 from 1541 kcal/24h to 1522 kcal/24h (p=0.64).

### 3.3 Thyroid hormon levels, resting energy expenditure and heart rate

**Figure 3:**
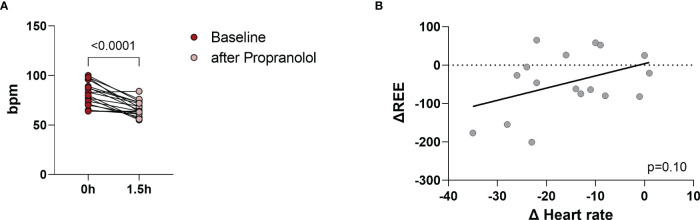
Pulse rate was measured before interventional visit and monitored every 30 minutes after administration of propranolol. Pulse reate decreased significantly (p<0.0001) from 81 ± 12 bpm (baseline) to 67 ± 7.6 bpm (after propranolol) **(A)**. The correlation between the change in heart rate (ΔHR) and the change in REE (ΔREE) was r=0.41, R^2^ = 0.17 and p=0.10 **(B)**.

Propranolol acutely lowers HR (19). The maximum plasma concentration of propranolol is reached within 60 to 90 minutes after oral intake (11). HR decreased significantly from baseline (81 ± 12 bpm) to 67 ± 7.6 bpm at 90 minutes (p<0.0001, **Figure 3A**) indicating that both dosage and absorption of propranolol were appropriate. We tested whether the change in heart rate (ΔHR) correlated with the change in resting energy expenditure (ΔREE) and thus could explain the slight reduction in REE. ΔHR and ΔREE were positively associated (R^2^ = 0.17) but the correlation was not significant (p=0.10, **Figure 3B**). In addition, we performed the statistical analysis separately for both groups (group 1: only treatment, group 2: treatment and control). They were similar in relation to HR, REE and TH levels (see **Table 1**).

**Figure 4:**
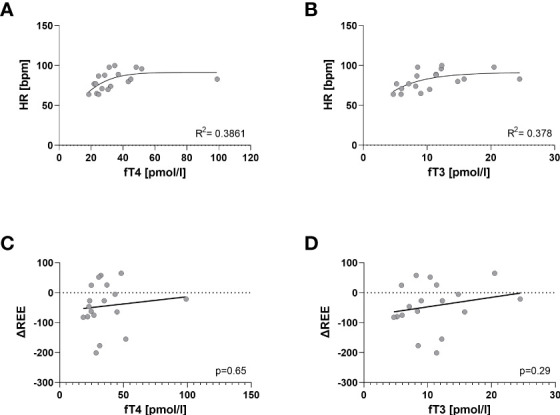
Influence of thyroid hormon levels on HR and ΔREE. Non-Linear Regression shows a positive correlation between HR and thyroid hormone levels; fT4 (R^2^ = 0.39, p=0.026) **(A)** and fT3 (and R^2^ = 0.38, p=0.029) **(B)**. ΔREE did not correlate with level oft fT4 (p= 0.65) **(C)** or fT3 (p= 0.29) **(D)** respectively. The degree of hyperthyroidism did not affect the response of REE to propranolol.

The levels of fT4 and fT3 were positively associated with HR (R^2^ = 0.39, p=0.026 and R^2^ = 0.38, p=0.029, **Figures 4A, B**). However, the degree of hyperthyroidism did not affect the response of REE to propranolol (**Figures 4C, D**).

### 3.4 Core body temperature and skin temperature

**Figure 5:**
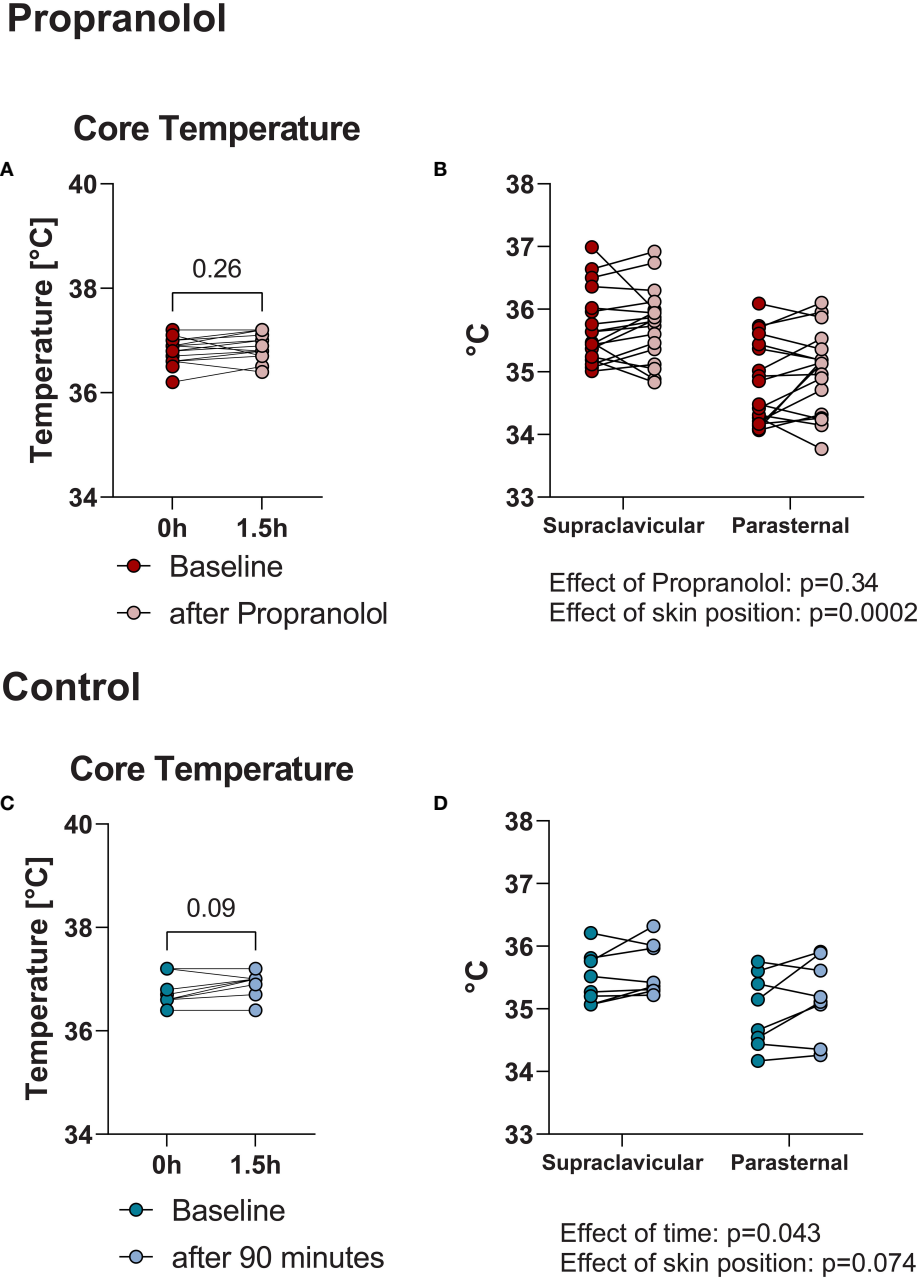
Mean tympanic temperature did not change after administration of propranolol: baseline 37.0 ± 0.25°C and 90 minutes after propranolol: 37.0 ± 0.27°C (p=0.26) **(A)**. Control visit (90-minute resting phase): baseline: 37.0 ± 0.29°C, after 90 minutes: 37.0 ± 0.24°C (p= 0.09) **(C)**. Mean supraclaviculare skin temperature compared to parasternal skin temperature as a reference. Supraclavicular skin temperature was higher than parasternal (effect of supraclavicular vs. parasternal position, p=0.0002) but did not significantly change after propranolol (effect of propranolol p=0.34) **(B)**. Similar results were observed during the control visit **(D)**.

It is known, that hyperthyroidism increases core body temperature in rodents (20). In hyperthyroid humans core body temperature is often markedly elevated in the setting of thyroid storm (21) but usually normal in clinically stable hyperthyroid outpatients (18). We wondered whether the acute dose of propranolol affected body temperature. Therefore, we assessed core body and skin temperature. The mean tympanic temperature, as a surrogate marker of core body temperature, did not change after a single dose of propranolol (baseline: 37.0 ± 0.25°C, 90 minutes after propranolol: 37.0 ± 0.27°C, p=0.26, **Figure 5A**). In the control visit, the results were comparable (baseline: 37.0 ± 0.29°C, 90 minutes after resting period: 37.0 ± 0.24°C, p= 0.09, **Figure 5C**).

BAT is mainly localized in the supraclavicular region (22) and measurements of supraclavicular skin temperature have previously been used as a surrogate marker of BAT activity (23). Therefore, we assessed whether skin temperature in the supraclavicular region decreased as a marker of lower BAT activity in response to propranolol. We compared those measurements to the parasternal skin temperature as a reference because this skin area is centrally located but not close to BAT depots. Supraclavicular skin temperature was higher than the parasternal region: 35.7 ± 0.6°C vs. 34.8 ± 0.7°C before and 35.8 ± 0.6°C vs. 35.0 ± 0.7°C 90 minutes after propranolol (effect of supraclavicular vs. parasternal position, p=0.0002, effect of propranolol p=0.34, **Figure 5B**). Results were similar during the control visit: supraclavicular baseline 35.5 ± 0.4°C, parasternal 35.0 ± 0.6°C and 35.6 ± 0.4 vs. 35.2 ± 0.6 after 90 minutes (p=0.074 for skin position and p=0.043 for time, **Figure 5D**).

## 4 Discussion

Hyperthyroidism elevates patients’ REE and significantly increases their oxygen consumption. Especially in the setting of thyroid storm or in patients with concomitant cardiopulmonary disease it is highly desirable to acutely reduce REE. Beta-adrenergic blockade with propranolol or other β-blockers is recommended to quickly alleviate the symptoms of hyperthyroidism (24). In the present study, we investigated the acute effect of propranolol on REE in patients with overt hyperthyroidism. We show here that an acute dose of 80 mg of propranolol does not reduce REE in a clinically meaningful way.

Theoretically the amount of propranolol given in our study could have been insufficient. In a previous trial propranolol effectively reduced REE when given to patients suffering from hyperthyroidism for a period of four weeks. Patients received a cumulative daily amount of 160 mg to 320 mg, divided into four single doses which translates into similar plasma levels as a single dose of 80 mg (12). Additionally, thyroid dysfunction can influence disposition of drugs. In hyperthyroid patients, absorption and metabolism of numerous pharmaceuticals are altered (25). Propranolol shows variability in plasma concentrations in healthy as well as in thyrotoxic patients (26–28). This could possibly be due to the increased hepatic blood flow and activity of the liver to metabolize the drug (28, 29). Thus, the dose required to reach therapeutic levels is variable and possibly higher in hyperthyroid patients. In our study propranolol significantly reduced heart rate indicating that plasma levels were sufficient to inhibit β_1_-ARs.

Patients underwent calorimetry in a fasted state and predominantly metabolized fatty acids (FAs) as indicated by a respiratory quotient (RQ) close to 0.7. During the control visit the RQ declined further, in line with increased use of FAs. However, after intake of propranolol, the RQ remained stable implying that propranolol slowed lipolysis by blocking β_2_-ARs. Taken together, these points indicate that the amount of propranolol given in our study was sufficient to elicit clinically relevant effects.

BAT is a major target of TH and metabolically active in hyperthyroidism even in the absence of a cold stimulus (8). Therefore, we hypothesized that BAT contributes significantly to REE and that its activity can be reduced by propranolol. The β_3_-AR has been described as the main AR facilitating the activity of brown adipocytes upon stimulation by norepinephrine. Propranolol is generally viewed as a non-selective β-AR antagonist which primarily concerns its effect on β_1_- and β_2_-ARs (30). Its antagonistic effect at the β_3_-AR has been questioned in the past (31), but it is clearly more capable to inhibit the β_3_-AR than other β-blockers in clinical use (30). In human volunteers who underwent mild cold exposure, propranolol did not reduce cold-induced thermogenesis and the authors speculated that this might be due to limited inihibition of the β_3_-AR (32). However, in an animal model propranolol at a dosage of 5 mg/kg body weight was able to abrogate the effect of a selective β_3_-AR-agonist on glucose uptake into BAT as determined by ^18^F-FDG-PET/CT (33). Moreover, interventional studies in euthyroid patients undergoing routine ^18^F-FDG-PET/CT scans showed that prior administration of propranolol minimizes FDG uptake into BAT as compared to control (15–17). Most of these studies were performed with a single dose of 20 to 80 mg propranolol. Recently, it has been suggested that not the β_3_-AR but the β_2_-AR facilitates BAT activation in humans (34). In this case, propranolol should be able to inhibit adrenergic signaling to BAT as well.

TH are crucial for BAT differentiation and activity and are required for mitochondriogenesis and the regulation of UCP1 in response to cold exposure (35). Thyroidectomized rats have a threefold reduction in UCP1 levels, which leads to an insufficient thermogenic response to cold (36). Replacing thyroxine in physiological amounts normalized UCP1 expression and prevented hypothermia (37). Additionally, TH regulates the sensitivity of tissues to sympathetic stimulation, which can induce thermogenic adipocytes in WAT in the hyperthyroid state (38, 39). The TH mediated effect in adrenergic responsiveness of BAT is still not fully understood. Some early studies showed a higher expression of β-AR in BAT due to TH, but the results are contradictory. Mild to moderate hyperthyroidism leads to a decreased adrenergic responsiveness of BAT probably due to the increased thermogenesis in other tissues, while severe hyperthyroidism leads to a increased BAT activation (8, 35). On the other hand, without the SNS drive, TH alone cannot upregulate UCP1 (35, 40). Under warm ambient conditions, BAT activity as determined by ^18^F-FDG-PET/CT is higher in hyperthyroid humans than in euthyroid controls. However, it does not reach the levels elicited by mild cold exposure which are several fold higher (8, 41).

Recent data from experiments in rodents indicate that the increase in REE and body temperature induced by high levels of thyroxine are centrally mediated and independent of UCP1 (20) suggesting that muscle and possibly other tissues contribute significantly to the higher metabolic rate. REE is the sum of thermogenesis in all tissues of the human body. While BAT is thermogenically highly active, it comprises only a small amount of body mass, especially in comparison to skeletal muscle. Muscle energy metabolism has been assessed by magnetic resonance spectroscopy in healthy volunteers who had been rendered mildly hyperthyroid by supraphysiological supplementation of T3 for three days. Coupling between mitochondrial respiration and ATP regeneration was reduced and whole body REE was increased (42). In patients with resistance to thyroid hormone associated with mutations in thyroid hormone receptor β (THRB) skeletal muscle is chronically exposed to mildly elevated TH levels. These individuals exhibit increased REE and increased tricyclic acid cycle flux due to inefficient mitochondrial coupling (43). Calcium cycling involving the sarcoplasmatic reticulum Ca^2+^-ATPase (SERCA1) constitutes a major mechanism of futile cycling within skeletal muscle (44). Importantly, the expression of SERCA1 is transcriptionally controlled by TH (4). Indeed, muscle biopsies in hyperthyroid individuals revealed higher amounts of skeletal muscle Ca^2+^-ATPase and Na^+^-K^+^-ATPase which correlated positively with EE and TH levels (45). In line with these studies, fasting glucose uptake into skeletal muscle was significantly elevated in hyperthyroid subjects as determined by ^18^F-FDG-PET/CT (8, 46). This underscores that skeletal muscle may substantially contribute to the higher REE in hyperthyroidism.

β_2_-AR signaling increases skeletal muscle lipid oxidation and thermogenesis (47) as well as mitochondrial biogenesis (48). These effects of catecholamines can be blunted by β-AR-antagonists (49). In the absence of an adrenergic stimulus, however, administration of propranolol did not reduce thermogenesis: Propranolol given at a dose of 160 mg daily to healthy volunteers did not reduce sleeping metabolic rate, which is comparable to REE (32).

In addition, increased metabolism within the liver may contribute to the elevation of REE in hyperthyroidism. Investigations in isolated hepatocytes from hyperthyroid rats revealed an increased mitochondrial proton leak as well as a higher ATP consumption as a source of stimulated thermogenesis (50). Additionally, the energy demand of myocardial tissue increases with HR in a linear fashion (51). Propranolol clearly reduced HR, which is in accordance with in previous studies (19, 52). In our trial, it was reduced by approximately 20% on average which would explain a reduction in total REE of approximately 30 to 40 kcal/d (53) which could actually represent the small change in REE we observed.

Although skin temperature in the supraclavicular region where BAT is mainly located (23, 54), was significantly higher than in the parasternal region, it did not change after administration of propranolol. This suggests, that propranolol actually has only a minimal effect on the increased BAT activity in hyperthyroid patients.

β-blockers and especially propranonol can have a direct effect on TH metabolism, especially by the conversion of T4 to T3 (14, 55, 56). When given over a period of four weeks propranolol could reduce REE in hyperthyroid patients and this effect is likely be due to reduced levels of T3 (12). As T3 has a plasma-half life of approximately 19 hours, a single dose of propranolol will therefore not lead to noticeable effect on REE by lowering levels of T3.

Our study is the first to evaluate the effects of propranolol on REE in hyperthyroid patients. Strengths of our study comprise a prospective design and a cohort representing the clinical reality of hyperthyroid patients. Our conclusions on the effect of propranolol on BAT activity are limited by the fact that we did not employ ^18^F-FDG-PET/CT to visualize and assess the glucose uptake into the tissue. Moreover, we only had a control visit in eight of the eighteen analyzed patients. However, from a practical perspective this is unlikely to alter our main finding.

## 5 Conclusion

In hyperthyroid patients a single dose of propranolol reduced heart rate substantially, but REE diminished only marginally probably due to reduced myocardial energy consumption. Our data speak against a relevant contribution of BAT to the increased REE in hyperthyroidism.

## Data availability statement

The datasets used and/or analyzed during the current study are available from the corresponding author on reasonable request. The request will be judged by an independent committee at the Department of Clinical Research of the University Hospital Basel to ensure that legal obligations are fulfilled.

## Ethics statement

The studies involving human participants were reviewed and approved by medical ethics committee of the University of Basel (ID EKNZ 2017-02044). The patients/participants provided their written informed consent to participate in this study.

## Author contributions

JRS: contributed to writing of the study protocol, performed experiments, analyzed data, and wrote the first draft of the manuscript. RCL: performed experiments. JGWF: performed experiments. FB: performed experiments. CIM: performed experiments, analyzed data. MJB: conceived the study, wrote first draft of study protocol, analyzed data, wrote the manuscript together with JRS. All authors critically reviewed and approved of the manuscript.
