# Supplementary material for: Acute effect of propranolol on resting energy expenditure in hyperthyroid patients
Source: Front Endocrinol (Lausanne). 2023 Jan 19;13:1026998. doi: 10.3389/fendo.2022.1026998 (PMC9892445; doi:10.3389/fendo.2022.1026998)
Supplement: Supplementary file 1 [file Table_1.docx]

# Supplementary Table 1: Hyperthyroid Symptom Scale (1)

| **Hyperthyroid Symptom Scale** | Score |
| --- | --- |
| **Nervousness**  0 absent  1 anxious only with stress  2 occasionally anxious at rest  3 often anxious, difficulty working or concentrating  4 states freely that feels “very nervous most of the time” |  |
| **Sweating**  0 only with activity  1 at rest but only in warm temperatures  2 at rest in temperate climates, mainly involving the hands and intertriginous zones  3 at rest involving many body areas  4 profusely diaphoretic almost constantly |  |
| **Heat intolerance**  0 normal temperature tolerance  1 periods of feeling warmer than those in the same room  2 significant difficulty with heat, requiring air conditioner constantly in the summertime  3 excessive difficulty with heat even in temperate climates  4 extreme difficulty with heat, does not feel comfortable even in cold weather as evidenced by lack of need for warm clothing or bed covers |  |
| **Hyperactivity**  0 normal activity level  1 increased activity level, increased productivity  2 increased productivity; decreased sleep time  3 performs some purposeless activity  4 frequent episodes of purposeless activity; unable to sit still during examination |  |
| **Tremor: Examination of outstretched hands**  0 absent  1 barely perceptible  2 tremor demonstrated readily on examination  3 marked tremor but able to perform fine motor skills  4 hands shake excessively, difficulty performing fine motor skills |  |
| **Weakness**  0 normal strength  1 subjective weakness but with normal exercise tolerance  2 decreased exercise tolerance to near maximal activity  3 decreased tolerance to stair vlimbing or arising from chair  4 extreme weakness such that patient can barely lift objects or walk up stairs |  |
| **Hyperdynamic precordium**  0 normal precorium activity and apical impulse  1 tachycardia, 90 beats per minute with normal apical impulse  2 tachycardia, 90 beats per minute with increased apical impulse  3 tachycardia, 110 beats per minute with increased apical impulse  4 tachycardia 110 beats per minute, apical impulse and carotid upstroke both increased, systolic outflow murmur |  |
| **Diarrhea**  0 1 bowel movement (BW) per day; formed stool  1 2-4 formed BMs per day  2 1-4 loose stools per day  3 4 formed BMs per day  4 4 loose stools per day |  |
| **Appetite**  0 appetite normal, no weight loss  1 appetite normal, weight loss  2 appetite increased, no weight loss  3 appetite increased, weight loss  4 appetite decreased, weight loss |  |
| **Assessment of daily function (degree of incapacitation)**  0 normal (none)  1 minimal impairment (10%)  2 mild impairment (30%)  3 moderat impairment (60%)  4 Severe impairment (90%) |  |
