# Supplementary material for: Acute effect of propranolol on resting energy expenditure in hyperthyroid patients
Source: Front Endocrinol (Lausanne). 2023 Jan 19;13:1026998. doi: 10.3389/fendo.2022.1026998 (PMC9892445; doi:10.3389/fendo.2022.1026998)
Supplement: Supplementary file 2 [file Table_2.docx]

**Supplementary Table 2:** Diagnosis of hyperthyroidism, therapy and duration of therapy. 15 patients were on thyrostatic therapy at the time of study inclusion. The median of treatment duration was 8 days.

| **Diagnosis** | **Thyrostatic therapy and Dosage/d**  (at time point of Participation) | **Duration of therapy in days**  (until the time of Participation) |
| --- | --- | --- |
| Graves' disease | Carbimazol, 40mg | 8 |
| Graves' disease relapse | Carbimazol, 45mg | 3 |
| Graves' disease | Carbimazol, 25mg | 2 |
| Graves' disease | Carbimazol, 30mg | 6 |
| Overtreatment | - | - |
| Graves' disease relapse | No therapy | - |
| Graves' disease | No therapy | - |
| Graves' disease | Carbimazol, 10mg | 5 |
| Graves' disease | Carbimazol, 45mg | 14 |
| Graves' disease | Carbimazol, 10mg | 6 |
| Graves' disease | Carbimazol, 20mg | 26 |
| Subacute thyreoiditis | No therapy | - |
| Graves' disease | Carbimazol, 20mg | 7 |
| Graves' disease | Carbimazol, 15mg | 19 |
| Graves' disease | Carbimazol, 20mg | 8 |
| Graves' disease | Carbimazol, 30mg | 10 |
| Graves' disease | Carbimazol, 20mg | 11 |
| Subacute thyreoiditis | Carbimazol, 20mg | 8 |
| Graves' disease | Carbimazol, 20mg | 5 |
